# Experiences of Health Research Data Sharing Among Researchers in Sub-Saharan Africa: Cross-Sectional Study

**DOI:** 10.2196/69411

**Published:** 2025-10-23

**Authors:** Felix Sukums, Bernard Ngowi, Rebecca Chaula, Kim L Weiszhar, Akili Kalinga, Olena Ivanova, Clarissa Prazeres da Costa, Achim Hoerauf, Sundeep Sahay, Emilia Virginia Noormahomed, Linda B Debrah, Chummy Sikasunge, Helena Ngowi, Andrea S Winkler

**Affiliations:** 1 Digital Health Unit, School of Public Health and Social Sciences, Muhimbili University of Health and Allied Sciences, 9 United Nations Road, Upanga West, Dar es Salaam, United Republic of Tanzania; 2 Mbeya College of Health and Allied Sciences, University of Dar es Salaam, Mbeya, United Republic of Tanzania; 3 Muhimbili Medical Research Centre, National Institute for Medical Research, Dar es Salaam, United Republic of Tanzania; 4 Directorate of ICT, Muhimbili University of Health and Allied Sciences, Dar es Salaam, United Republic of Tanzania; 5 Department of Neurology, TUM University Hospital, and Center for Global Health, School of Medicine and Health, Technical University of Munich (TUM), Munich, Germany; 6 National Institute for Medical Research, Dar es Salaam, United Republic of Tanzania; 7 Institute of Infectious Diseases and Tropical Medicine, LMU University Hospital, LMU Munich, Munich, Germany; 8 German Centre for Infection Research (DZIF), Partner Site Munich, Munich, Germany; 9 Institute of Medical Microbiology, Immunology and Parasitology (IMMIP), University Hospital Bonn, Bonn, Germany; 10 Department of Informatics, University of Oslo, Oslo, Norway; 11 Faculty of Medicine, Eduardo Mondlane University (UEM), Maputo, Mozambique; 12 Mozambique Institute for Health Education and Research (MIHER), Maputo, Mozambique; 13 School of Medical Sciences, Kwame Nkrumah University of Science and Technology, Kumasi, Ghana; 14 School of Veterinary Medicine, University of Zambia, Lusaka, Zambia; 15 Department of Veterinary Medicine and Public Health, Sokoine University of Agriculture, Morogoro, United Republic of Tanzania; 16 Department of Community Medicine and Global Health, Institute of Health and Society, University of Oslo, Oslo, Norway; 17 Department of Global Health and Social Medicine, Harvard Medical School, Boston, MA, United States

**Keywords:** health research data, research data management, research data sharing, health research network, ICT, digital research data repositories, sub-saharan Africa, information and communication technology

## Abstract

**Background:**

Digital platforms play a vital role in improving the availability and access to health research outputs, enhancing the engagement of policy makers and practitioners in the research processes. Despite their potential, it needs to be explored how digital platforms are used to manage and share health research datasets and publications, and to translate research findings among health networks or institutions in sub-Saharan Africa (SSA).

**Objective:**

This study aimed to assess the practices of health research data management, including sharing among researchers and their support staff within 3 large research networks for health innovations in SSA.

**Methods:**

A cross-sectional mixed methods survey was conducted across 3 research networks in SSA, showing experiences of sharing research data using digital platforms among researchers of 3 large research and innovation networks in SSA and affiliated institutions in the Global North. A total of 160 respondents completed a self-administered web-based questionnaire, and following data cleansing, the survey data were analyzed using both descriptive and inferential statistics.

**Results:**

Most respondents (91/160, 56.9%) used electronic data collection tools to collect research data. Almost half (79/160, 49.4%) of the respondents have a digital research data management platform. More than half of the respondents shared their research datasets (102/160, 63.8%), and 61.3% (98/160) shared research findings with the research community through different channels. Furthermore, most respondents shared their research datasets and research outputs through institutional data repositories (42/160, 26.1%), scientific conferences (123/160, 76.9%), and journal articles (110/160, 68.8%). This study found that parameters such as sex, professional category (health professional, information and communication technology professional, and data managers), and the role (researcher or student) influence health research data sharing within the community. The results show that the roles of the individual have the strongest association with the sharing of research datasets, followed by years of experience in research, then sex, and profession. Females were less likely to share their research datasets than males. Data managers and information and communication technology professionals exchanged datasets less frequently in the professional group, and the researcher’s role was statistically significant in sharing research datasets.

**Conclusions:**

This study demonstrates that most researchers share research datasets and outputs through various channels. It was further found that digital platforms were essential in managing and sharing research datasets and publications since more than half (85/160, 53.1%) of the respondents have and use digital platforms. In addition, the study identified factors that influenced researchers’ practices of sharing research datasets and publications. Furthermore, key gaps limit the sharing of these research datasets, including inadequate infrastructure, insufficient African dataset sharing platforms, a lack of institutional policy, and limited skills to use available platforms.

## Introduction

The World Health Organization (WHO) defined knowledge translation as the exchange and application of knowledge by relevant stakeholders to accelerate the benefit of global and local innovation in strengthening health systems and improving people’s health [1]. Research findings need timely translation into practice and policy [2,3]. Translation of research findings may lead to evidence-based practice guidelines, adoption of technologies, or health care delivery models with proven value, and a change in practice and behaviors [3,4]. Thus, there is a need to establish substantial programs of health research data sharing for improvement in health care delivery and policy formulation [4].

Data sharing and its significance connect to research findings. Findable, accessible, interoperable, and reusable (FAIR) principles are the foundation for effective data sharing [5]. FAIR guiding principles for scientific data management support a vision for data sharing by becoming more findable, accessible, interoperable, and reusable, whereby these principles were intended to apply to many kinds of digital assets or content types [6,7].

Several systematic reviews have assessed barriers and facilitators to the use of research evidence in health decision-making [8-11], pointing out the need for timely access to good quality, relevant, and applicable research evidence, collaborations with decision makers, and building relationships and skills. Interventions to boost the translation of research findings into policy and practice include formats such as evidence briefs, issue briefs, policy briefs, press releases, web portals, consultations, deliberative dialogs, and workshops [12]. Literature shows that one of the best practices for research data sharing within the scientific field is using digital data repositories [13-15].

Data sharing platforms are systems that enable the collection, storage, and reuse of research datasets across all disciplines. Despite global progress in the development of digital platforms for research data sharing, sub-Saharan Africa (SSA) remains significantly underrepresented in the open science ecosystem. Existing platforms, such as CKAN, Zenodo, Figshare, Dryad, and Dataverse, have played a pivotal role in facilitating open access to research data by enabling data publication, discoverability, and reuse through standardized metadata, persistent identifiers, and open licensing models [16,17]. Collaborative environments, like GitHub and Kaggle, also support data sharing alongside reproducible analytics and community engagement. Furthermore, initiatives such as OpenAIRE and the European Open Science Cloud illustrate how regional efforts can institutionalize open science practices [18]. However, in the context of SSA, participation in and benefit from such platforms remain limited due to infrastructural barriers, uneven internet access, limited institutional support for data management, and capacity constraints in data stewardship [8,19]. While platforms like the African Open Science Platform and the DataFirst repository at the University of Cape Town signal regional progress, they remain relatively few and often underresourced [20,21]. This imbalance restricts the visibility, accessibility, and global integration of African research outputs, exacerbating existing inequalities in knowledge production and dissemination. Therefore, addressing these disparities through investment in regional platforms, policy alignment, and capacity building is essential to ensure equitable participation in the global open science movement.

Despite global advances in open science, SSA continues to face significant barriers to research data sharing. High-income countries benefit from strong digital infrastructure, FAIR data policies, and established repositories that promote open access and reuse [5,22]. In contrast, the African region struggles with poor connectivity, limited funding, inadequate data stewardship skills, and fragmented policies [19,23,24]. Concerns around data ownership, language diversity, and lack of awareness further hinder participation [8,25,26]. While platforms like European Open Science Cloud, Zenodo, and Dryad support global data sharing [27,28], similar efforts in Africa, such as African Open Science Platform and DataFirst, remain underresourced. Bridging this gap requires investment in infrastructure, harmonized policies, and capacity building tailored to local contexts.

Even though data sharing in science is not yet practiced routinely [29], there has been an increasing emphasis on the governance and storage of research data in recent times. Accordingly, research stakeholders and funders more often recognize the value of scientific data [29,30]. Open Science, including Open Data, is focused mainly on promoting the sharing of scientific research work among stakeholders [15,29-32]. It is being advocated to ensure open access to research data for further study and for informed decision-making among stakeholders, including policy makers, practitioners, librarians, data managers, and research funders. Furthermore, the WHO emphasizes that the global scientific community promotes the culture of sharing datasets even before publication to validate the findings and data reuse, as well as early translation of research results for health practices and policy [1]. Consequently, new guidelines entitled “Sharing and reuse of health-related data for research purposes: WHO policy and implementation guidance” were published in 2022 [1]. The European Commission has also been actively promoting making research data more accessible for years. A prominent and successful example of this endeavor is the European COVID-19 data platform launched in 2020 to combat the pandemic worldwide [33].

Data sharing is being advocated to be in electronic formats for easier management and accessibility [34]. Digital platforms or data repositories help create online spaces within which the producers and users of information can share resources and learn [8,26]. Research data shared among stakeholders includes various types and formats. These consist of quantitative and qualitative datasets, such as survey responses, clinical data, patient records, and demographic surveillance, along with real-time data from human participants, health system data, epidemiological data, and human biological specimens. Shared data may also comprise images, technical reports, financial documents, models, and digital health data from wearables and eHealth apps [7,17,25,26,35].

Despite the rich potential of platforms to enable the sharing of research findings between researchers, policy makers, and practitioners, their value has not been effectively realized, particularly in the context of health research in low- and middle-income countries (LMICs). Many issues surrounding digital scientific data sharing, such as legal, ethical, cultural, and intellectual rights, remain unknown. New problems and questions are emerging as the old ones are increasingly addressed, but technical challenges still hinder open data sharing on a broader scale.

Leading publishers, such as Springer Nature, Elsevier, Taylor and Francis, and Wiley, have introduced and started encouraging authors to provide quality research datasets through the publisher’s digital platforms or other publicly available platforms, for example, a general-purpose data repository for easier accessibility of the data to a broad audience [34,36,37]. In 2016, Wiley surveyed 4600 authors from 112 countries to get insights into research data sharing. The survey indicated that 69% (n=3174) of the authors shared data. Barriers to data sharing included intellectual property or confidentiality issues, ethical concerns, fear of data misinterpretation or misuse, and fear of scooping [37,38]. Only 92 respondents (authors) were from SSA, and many countries, such as Tanzania and Ghana, were not represented.

Overall, as transparent data sharing has become increasingly a requirement for good scientific practice at different levels, little is known about how digital platforms have recently been used to manage and share health research data and publications within SSA.

Health research consortia, such as CYSTINET-Africa, TAKeOFF, and TB Sequel, are spearheading efforts to co-develop a best-practice model for the early digital translation of disease-related research data across 5 health innovation networks in SSA. Simultaneously, leading academic publishers—including Springer Nature, Elsevier, Taylor & Francis, and Wiley—have begun requiring authors to submit quality-assured research datasets via their own digital platforms or through publicly accessible repositories to enhance data discoverability and reuse [11-13]. A 2016 Wiley survey involving 4600 authors from 112 countries revealed that while 69% (3174/4600) of respondents shared data, significant barriers remained, such as intellectual property concerns, ethical restrictions, fears of misinterpretation, and risks of being scooped [13]. Notably, only 92 of those respondents were from SSA, with key countries such as Tanzania and Ghana unrepresented. This highlights a pressing need to assess and address regional gaps. Accordingly, this study aims to examine existing practices and future needs regarding the timely sharing of research data related to infectious diseases, neglected tropical diseases, noncommunicable diseases, and One Health across subnational and national levels within the 5 targeted research networks. The findings will inform the development of long-term, scalable digital data-sharing prototypes that are aligned with the needs of researchers, policy makers, and stakeholders. These platforms are intended to enhance accessibility to research outputs and facilitate the integration of evidence into policy and practice, while ensuring alignment with both user requirements and reporting mandates at national and subnational levels.

This study aimed to assess the practices of health research data management, including sharing among researchers and their support staff within 3 large Research Networks for Health Innovations in SSA funded by the German Federal Ministry of Education and Research (BMBF). We specifically assessed the types of data shared, the methods of sharing, the timing of sharing, and the recipients of the data. In addition, the study assessed how researchers disseminated research data for reuse by other researchers and how they shared research outputs to facilitate translation for informing policy practice.

## Methods

### Study Setting

**Figure 1.**
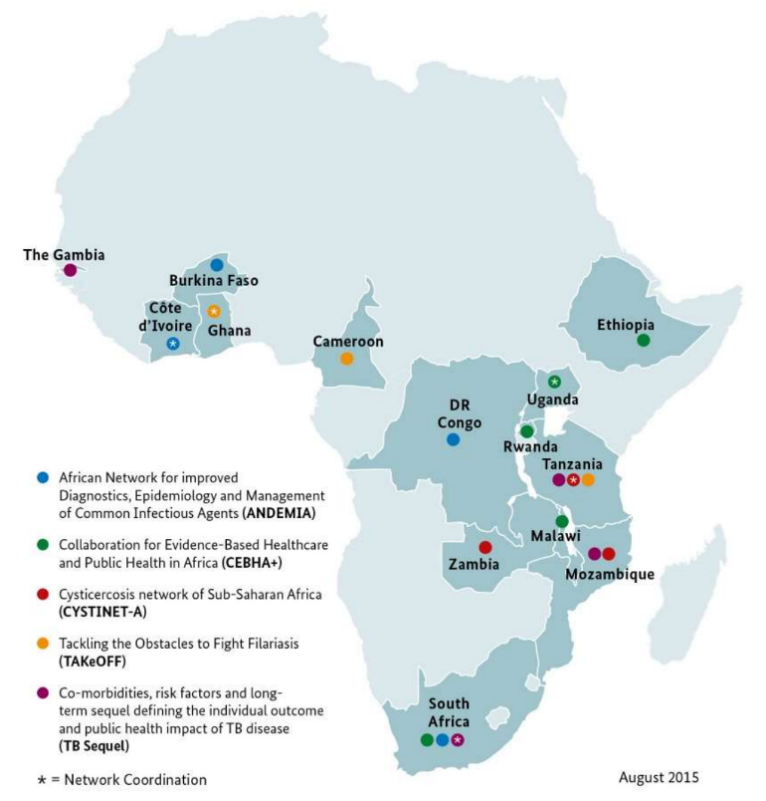
Geographical locations of the supported health networks [39]. TB: tuberculosis.

The respondents of this study were researchers, including postgraduate students, and research support staff of 3 BMBF-funded Research Networks for Health Innovations in SSA, namely CYSTINET-Africa, TAKeOFF, and TB Sequel [39]. The partner institutions are located in different SSA countries (Figure 1). Participating institutions from Germany were also included in the study. Partner institutions conducted research in various areas, including infectious diseases, neglected tropical diseases, noncommunicable disease, One Health, and digital health.

### Study Design

This study used an online cross-sectional design to assess the experiences of researchers and support staff with research data management and sharing at subnational and national levels and to determine factors hindering the use of digital platforms for this purpose within SSA’s 3 health networks.

### Ethical Considerations

Ethical clearance was sought from the National Health Research Ethics Review Committee of the National Institute for Medical Research (NIMR) in Tanzania (ethical clearance certificate NIMR/HQ/R.8a/Vol.IX/3836). Verbal permission to implement this research was obtained from the coordinators of each research network. Respondents to the self-administered internet-based questionnaire through Google Forms agreed to participate in this study through informed consent, which was attached as the first section of the questionnaire. Respondents were asked to fill in the questionnaire after reading and accepting the contents of the consent form. No personally identifiable information about respondents was collected. Research data were kept secure and confidential by 2 researchers (FS and BN) and 1 data manager at NIMR in Tanzania. There was no compensation provided to participants.

### Questionnaire Development

The questionnaire used in this study was developed through a structured and iterative process designed to capture relevant practices, challenges, and needs related to research data sharing across health research networks in SSA. The development started with a review of existing literature and validated instruments on data sharing practices, open science frameworks, and digital health governance. Key domains were identified and aligned with the study objectives, covering topics such as research data management, research data management platforms, dissemination of research data, general needs and requirements for data sharing platforms, and stakeholder engagement. Experts from the participating research networks—CYSTINET-Africa, TAKeOFF, and TB Sequel—were consulted to ensure contextual relevance and content validity. A draft version of the questionnaire was then pretested with a small sample of 15 researchers from the target networks to assess clarity, comprehensiveness, and length. Based on feedback from the pilot, minor revisions were made to improve wording, flow, and response options. The final instrument included both closed- and open-ended questions and was administered in English through an online platform to facilitate broad participation. The complete questionnaire is provided in Multimedia Appendix 1 for reference and transparency.

### Data Collection Tools and Techniques

A self-administered web-based questionnaire was developed using Google Forms and shared with study participants for completion. Data were collected using a nonprobability sampling method, in which participants were directly contacted and referred to the research network team by sharing the link. Each of the network coordinators provided the contact information from the network. The duration of data collection was initially planned for 2 months (September and October 2021) but was later extended to December 2021 due to delays in responses. A reminder message was sent every Friday of the week. The questionnaire was pretested among 15 researchers outside the 3 research networks to determine the ease of completing it and to ensure that the questions were clearly understood.

The experience gained from the pretest was used to improve the tools. Web-based questionnaire administration enables access to large and diverse populations and is an inexpensive, quick, and convenient approach to collecting data [40-42]. Furthermore, the questionnaire had mandatory fields for quality checks to ensure that all relevant fields were filled in before the data were submitted to a database.

The questionnaire explored practices of sharing raw research datasets and outputs to facilitate the early translation of research into policy and practice within the 3 networks. The questionnaire also examined the existence and use of digital platforms within the 3 health research networks to improve the early translation of research findings into practice and policy. The questionnaire collected researchers’ sociodemographic data, explored the existence of data or information systems and data sharing practices within the research projects, and assessed experience using research data management digital platforms and disseminating research data, including anonymized raw research datasets and outputs.

### Data Analysis

The quantitative data were transferred from the Google Forms server onto a Microsoft Excel file to verify data completeness and consistency and liaise with respective network coordinators. After extracting data from Google Drive, it was stored in a secure computer with a password, ready for cleaning and analysis. Researchers assessed and verified the data for quality before compiling it for analysis. Frequency tables were prepared to assist in data cleaning. The software developed by IBM, SPSS version 23.0, was used for univariate and bivariate analyses. Differences between categories were measured using chi-square or Fisher exact tests, and binary logistic regression was used to determine the factors that influence the sharing of research datasets.

After data collection, several variables were transformed to enhance analytical clarity and improve the robustness of inferential statistics. These transformations included recoding research network membership, categorizing age groups according to WHO standards, and standardizing variables such as sex, education level, professional role, and years of experience.

We explored factors (variables) that influence the sharing of research datasets and publications through different channels among the respondents. The binary logistic regression (crude odds ratio [cOR] and adjusted odds ratio [OR]) was used in this study since the dependent variable was dichotomous, involving 2 choices: whether the respondents shared their research datasets or outputs. In this study, the independent variables were all categorical.

### Measure the Importance and Associations of the Categorical Variables

Cramer V measures associations and importance for the categorical variables. The statistics range from 0 to 1, where 0 indicates no association, and 1 indicates a perfect association. A larger value for Cramer V suggests a stronger association between variables. It is derived from the chi-square test and provides a value that shows the strength of associations between 2 variables. In our case, we measure the importance of the independent variables, which include sex, profession, roles, and years of experience [43].

## Results

### Overview

Table 1 illustrates the sociodemographic characteristics of the respondents. The majority (98/160, 61.3%) were from 3 health innovation research networks of SSA, of which 31.3% (50/160) were from CYSTNET-Africa, and 63.1% (101/160) worked at universities. Slightly more than half were aged 40 years or younger (83/160, 52.2%), the vast majority resided in East African countries (130/160, 81.3%), and over half (108/160, 67.5%) had 1 to 10 years of experience in research-related work. Half of the respondents were researchers (81/160, 50.6%), and the highest education level was almost equal between Master’s and PhD levels (64/160, 40.1% and 77/160, 48.1%, respectively).

**Table 1.** Sociodemographic characteristics of the respondents (n=160).

Sociodemographic characteristics	Frequency, n (%)
Research network^a^	
The 3 networks	98 (61.3)
Others	62 (38.8)
Institution category	
University	101 (63.1)
Research Institution	35 (21.9)
Others	24 (15)
Age group (y)	
21‐30	17 (10.7)
31‐40	66 (41.5)
41‐50	56 (35.2)
51 and above	20 (12.6)
Sex	
Female	58 (36.3)
Male	101 (63.1)
Preferred not to mention	1 (0.6)
Highest level of education	
PhD degree	64 (40)
Master’s degree	77 (48.1)
Bachelor’s degree	19 (11.9)
Research experience (years)	
1‐5	71 (44.4)
6‐10	37 (23.1)
11 and above	52 (32.5)
Professional category	
Health researchers	114 (71.3)
ICT^b^ officers and data managers	27 (16.9)
Others	19 (11.9)
Roles	
Researchers	81 (50.6)
ICT and data managers	30 (18.8)
Postgraduate (PhD and Masters)	35 (21.9)
Others	14 (8.8)
Types of research data collected in the research platforms^a^	
Human health data	139 (86.9)
Animal health data	40 (25)
Environmental health data	27 (16.9)
Other-not specified data	12 (7.5)
Sources of research data^a^	
Community-based sources	115 (71.9)
Health facilities	108 (67.5)
Other sources	48 (30)
Means of collecting primary and secondary research data^a^	
Using paper-based forms	51.9 (31.9)
Using electronic data collection tools	91 (56.9)

a Multiple options applied.

b ICT: information and communication technology.

The study indicated that different types of research data are collected from various sources, including, but not limited to, communities, health facilities, villages, and subnational and national levels, covering a wide range of health domains. Both paper and electronic data collection and management tools were used. Most respondents (91/160, 56.9%) used electronic data collection tools to collect research data.

### Frequency of Sharing Research Outputs among Respondents

In total, 53.6% (112/209) indicated that they share or disseminate their research findings for early translation of research findings as per the project plan. In comparison, 41.6% (87/209) indicated that they would share their research output when the opportunity arises. Also, in 76.9% (123/160) and 68.8% (110/160) of the cases, participants responded to sharing their research outputs for early translations of research findings through scientific conferences and journal articles, respectively, as illustrated in Table 2, on which different means of sharing research datasets were reported. Furthermore, 26.1% (61/234) and 12% (28/234) of the respondents used institutional data repositories and cloud file sharing as the channel to share research datasets, as illustrated in Table 3.

**Table 2.** Proportion of respondents and their different channels of sharing research datasets.

Channels	Frequency, n (%)
Institutional data repository	61 (26.1)
Funder data repository	19 (8.1)
Discipline-specific data repository	13 (5.6)
Journal data repository	28 (12)
As supplementary material in a journal	25 (10.7)
External repository or general-purpose data repository (eg, Figshare, Zenodo)	7 (3)
Cloud file sharing (eg, Dropbox, Google Drive, and Blog or website)	27 (11.5)
I do not share research data beyond my fellow researchers	54 (23.1)

**Table 3.** Proportion of respondents and their different channels of sharing research outputs.

Means	Frequency, n (%)
Project website	44 (7.2)
Social media channels	45 (7.3)
Newsletters	39 (6.4)
Scientific conferences	123 (20)
Journal articles and other publications	110 (17.9)
Policy briefs	50 (8.1)
Feedback meetings with policy makers	49 (8)
Feedback meetings with health professionals	60 (9.8)
Feedback meetings with community members	51 (8.3)
Reports submitted to the ministry, region, and district officials	43 (7)

Furthermore, access to the existing digital research platforms to a wider audience was also reported as one of the limitations. About one-third (53/169, 31.4%) of the respondents indicated they could share their research datasets mostly with their fellow researchers within their research institution or research networks (32/169, 18.9%), as presented in Table 4.

**Table 4.** Stakeholders who had access to research datasets through different platforms (n=160).

Sharing raw research datasets	Frequency, n (%)
Other researchers within their (own) research institution	53 (31.4)
Other researchers within the (own) research network	32 (18.9)
Other researchers in the other health innovation research networks	8 (4.7)
Other researchers outside their institutions and the health innovation research networks	13 (7.7)
Health professionals or practitioners in general	17 (10.1)
Policy makers at the subnational level	10 (5.9)
Policy makers at the national level	14 (8.3)
Policy makers at the international level	5 (3)
Own research funders	17 (10.1)

Researchers were asked if their research project had a data transfer or sharing agreement or policy with other partners. The results show that 100% (N=160) of the ANDEMIA and TAKeOFF have sharing agreements with the partners of their research network, followed by CYSTINET-Africa (13/15, 86.7%), TB Sequel (7/9, 77.8%), and CEBHA+ (2/4, 50%). Also, research institutes (19/23, 82.6%), European countries (N=9, 100%), and South Africa (N=4, 100%) only share their data with partners within their research network.

More females share their data through their research partners than males. Graduates with experience between 1 and 5 years (17/19, 89.5%) share their research project through their partners in the research network.

We analyze the availability of data-sharing agreements among the research projects (Table 5). There was cross-sharing between networks and third parties through data-sharing agreements. However, there is no statistically significant difference in the availability of data-sharing agreements among the 3 groups, as indicated in Table 5.

**Table 5.** Availability of data sharing agreements among the research projects.

Independent variables	Availability of data sharing agreement with (n=61)			Fisher exact test
	Partners of their research network, n (%)	Partners of any of the health innovation research networks, n (%)	Third-party, n (%)	
Research network				0.11
3 networks	33 (84.6)	7 (17.9)	8 (20.5)	
Others	14 (63.6)	8 (36.4)	4 (18.2)	
Institution category				0.35
University	24 (66.7)	6 (16.7)	6 (16.7)	
Research Institute	19 (65.5)	5 (17.2)	5 (17.2)	
Others	4 (44.4)	4 (44.4)	1 (11.1)	

### Practices in Sharing Research Datasets and Publications Among Respondents

Table 6 shows respondents’ experiences in sharing research datasets and research outputs and the availability of digital research data management platforms. Almost half (79/160, 49.4%) of the respondents reported that they had digital research data management platforms, which include Afyadata, KoboCollect, DHIS2, DSpace, REDCap, and Open Data Kit, 63.8% (102/160) were sharing research datasets, and 46.3% (74/160) were sharing research publications via the channels. The presence of digital research data management platforms showed a statistically significant difference between the institution categories (*P*=.013), in which 51.9% (41/79) of the respondents from universities reported using digital research data management platforms. Furthermore, statistically significant differences in sharing research datasets and publications were found in respondents’ sex, professional category, years of research experience, and roles.

**Table 6.** Respondents’ characteristics in the context of management and sharing of research datasets.

Independent variables	Shared research datasets (n=102)		Shared research findings (n=98)		Had digital research data management platforms (n=79)	
	n (%)	*P* value	n (%)	*P* value	n (%)	*P* value
Research network		.40		.51		.27
3 networks	60 (58.8)		62 (63.3)		45 (57)
Others	42 (41.2)	36 (36.7)	34 (43)
Institution category		.89		.07		.01
University	63 (61.8)		63 (64.3)		41 (51.9)	
Research Institution	23 (22.5)	25 (25.5)	23 (29.1)
Others	16 (15.7)	10 (10.2)	15 (19)
Age group (y)		.10		.11		.44
21‐30	9 (8.8)		10 (10.3)		11 (14.1)	
31‐40	38 (37.3)	36 (37.1)	35 (44.9)
41‐50	43 (42.2)	34 (35.1)	23 (29.5)
51 and older	12 (11.8)	17 (17.5)	9 (11.5)
Sex		.01		.25		.95
Female	30 (29.4)		32 (33)		29 (36.7)	
Male	72 (70.6)	65 (67)	50 (63.3)
Highest level of education		.10		.15		.08
PhD	47 (46.1)		45 (45.9)		32 (40.5)	
Masters	45 (44.1)	43 (43.9)	34 (43)
Graduate	10 (9.8)	10 (10.2)	13 (16.5)
Professional category		.04		.79		.38
Health professionals	69 (67.6)		69 (70.4)		53 (67.1)	
ICT^a^ and data managers	16 (15.7)		16 (16.3)		16 (20.3)	
Others	17 (16.7)		13 (13.3)		10 (12.7)	
Roles		.001		.20		.44
Researchers	61 (59.8)		54 (55.1)		43 (54.4)	
ICT and data managers	14 (13.7)		19 (19.4)		17 (21.5)	
Postgraduates (PhD and Masters)	16 (15.7)		16 (16.3)		13 (16.5)	
Others	11 (10.8)		9 (9.2)		6 (7.6)	
Research experience (y)		.03		.54		.31
1‐5	38 (37.3)		41 (41.8)		31 (39.2)	
6‐10	29 (28.4)		22 (22.4)		22 (27.8)	
11 and above	35 (34.3)		35 (35.7)		26 (32.9)	

a ICT: information and communication technology.

By using the Cramer V test, it shows that sex had a Cramer V coefficient of 0.20, indicating a strong association between sex and the datasets sharing and publications; profession had a coefficient of 0.20 and also had a strong association between the profession and the datasets sharing and publications; roles had a Cramer coefficient of 0.30, indicating a very strong association between roles and the datasets sharing and publications compared with sex and profession. Years of experience had a Cramer coefficient of 0.21, suggesting a strong association between years of experience and sharing research datasets and publications. So, based on Cramer V coefficient, the most important variables in association with the outcome variable are roles (0.30), years of experience in research (0.21), sex (0.20), and profession (0.20).

### Factors Influencing Research Data Sharing via Digital Research Channels or Data Management Platforms

Table 7 illustrates the logistic regression analysis to determine factors influencing whether respondents shared their research datasets. As presented in Table 7, sex, professional category, and roles significantly influence dataset sharing among respondents.

**Table 7.** Logistic regression analysis on factors influencing the sharing of research datasets.

Variables	cOR^b^ (95% CI)	*P* value	aOR^c^ (95%)	*P* value
Research network				
3 networks	0.752 (0.385‐1.469)	.40	1.063 (0.464‐2.439)	.89
Others	Reference	Reference	Reference	Reference
Institution category				
University	0.829 (0.324‐2.121)	.70	0.656 (0.198‐2.174)	.49
Research Institute	0.958 (0.319‐2.876)	.94	1.123 (0.293‐4.296)	.87
Others	Reference	Reference	Reference	Reference
Age group (y)				
21‐30	0.750 (0.203‐2.770)	.67	1.970 (0.287‐13.544)	.49
31‐40	0.905 (0.327‐2.507)	.85	1.486 (0.328‐6.727)	.61
41‐50	2.205 (0.742‐6.550)	.16	2.921 (0.746‐11.429)	.12
51 and older	Reference	Reference	Reference	Reference
Sex				
Female	0.432 (0.220‐0.845)	.01	0.385 (0.160‐0.927)	.03
Male	Reference	Reference	Reference	Reference
Education				
PhD	2.488 (0.864‐7.165)	.09	3.904 (0.826‐18.459)	.09
Masters	1.266 (0.462‐3.469)	.65	2.658 (0.710‐9.949)	.15
Graduate	Reference	Reference	Reference	Reference
Professional category				
Health professionals	0.180 (0.040‐0.819)	.03	0.108 (0.019‐0.614)	.01
ICT^a^ and data managers	0.171 (0.033‐0.895)	.04	0.244 (0.038‐1.578)	.14
Others	Reference	Reference	Reference	Reference
Roles				
Researchers	0.832 (0.211‐3.283)	.79	1.011 (0.178‐5.743)	.99
ICT and data managers	0.239 (0.055‐1.032)	.06	0.165 (0.026‐1.043)	.06
Students	0.230 (0.054‐0.969)	.05	0.239 (0.042‐1.361)	.11
Others	Reference	Reference	Reference	Reference
Research experience (y)				
1‐5	0.559 (0.266‐1.177)	.13	0.814 (0.245‐2.704)	.74
6‐10	1.761 (0.665‐4.663)	.26	1.748 (0.441‐6.923)	.43
11 and more	Reference	Reference	Reference	Reference

a ICT: information and communication technology.

b cOR: crude odds ratio.

c aOR: adjusted odds ratio.

Before and after adjusting for additional parameters, as illustrated in Table 7, sex has a statistically significant impact on the sharing of research datasets. Specifically, females are 56.8% less likely to share their datasets than males (cOR 0.432, CI 0.220‐0.845; *P*=.01). This difference persists even after adjusting for OR of 0.385 (CI 0.160‐0.927; *P*=.03).

Health professionals are significantly less likely than others to share their research datasets, even after controlling for other variables, according to a study on the professional category that is statistically significant before and after adjustment for OR 0.180 (CI 0.019‐0.614; *P=*.01).

There is statistical significance in students’ probabilities of sharing their study datasets compared with other respondents (OR 0.23, CI 0.054‐0.969; *P*=.05).

The OR increased after controlling for other factors, leading to a 60.3% higher likelihood of sharing the datasets in 3 networks than others (OR 1.603, CI 0.464‐2.439; *P*=.89). However, the *P* value indicates no significant difference in both cOR and adjusted OR.

The institutional category (university, research institutions, and others) shows a statistically insignificant association in sharing research datasets. After adjustment, the values (OR 0.656, CI 0.198‐2.174; *P*=.49) indicate a 34.4% lower likelihood of dataset sharing.

## Discussion

### Principal Findings

Our results show that different types of data are mainly collected using electronic tools. Most data collected for research platforms are human health data from health facilities and the community. The study investigated the experiences of researchers sharing research data using digital platforms in 3 large research and innovation networks of SSA and affiliated institutions. It also explored the sharing of research datasets, the availability of data sharing agreements among the research projects, and the practices in sharing research datasets and publications among respondents under SSA’s 3 health innovation research networks.

Respondents used various digital platforms, such as KoboCollect and REDCap, for data management. The presence of such platforms was more common in universities (*P*=.01), which may have more advanced research infrastructures than other institutions. The effectiveness of these tools in managing and sharing large datasets has been widely documented, especially in global health research.

The results show that most of the researchers from the networks have agreements or policies for sharing research datasets with their partners. A study conducted by Waithira et al [21], on data management and policy, emphasizes that each institution should have a policy and agreement to ensure ethical and smooth data sharing and promote the sharing of datasets among researchers. From the independent variables, the role of the researcher was more important in sharing their datasets, followed by years of experience in research. However, it did not show statistical significance then, followed by the profession of the researchers, and finally, sex.

### Practice in Data Sharing

The results show that different people share research datasets and outputs in different ways. Researchers share data at different stages of the research process—both during and after research completion—through various channels, such as institutional repositories, journal publications, and scientific conferences.

Of the respondents, approximately half (112/209, 53.6%) communicated research findings in accordance with project plans, and a comparable percentage (87/209, 41.6%) shared their research outputs when possibilities presented themselves. The main means of disseminating research outputs were conferences (123/160, 76.9%) and journal articles (110/160, 68.8%), with 26.1% (61/234, due to multiple responses) and 33.8% (61/180) of respondents using institutional repositories and cloud platforms, respectively. Unlike in our study, a study by Khan and colleagues [7] found that data sharing and reuse were more common among experienced researchers. Their findings indicated that researchers with previous experience in reusing data were more likely to share their own data than those without such experience. On the contrary, Tenopir et al [44] showed differences among age groups, with younger respondents feeling more positively about data sharing and reuse yet providing less of their data than older respondents.

Also, most researchers use institutional repositories to share their datasets [7]. About one-third (53/169, 31.4%) of respondents said they had access to digital research platforms within their institutions, indicating a lack of widespread availability.

Ethical and responsible data sharing requires appropriate agreements, particularly for cross-border data flows, to ensure compliance with national and international data protection laws [25,26]. Our study assessed the availability of data-sharing agreements across research projects. While data were shared between networks and third parties through such agreements, some projects lacked formal agreements. However, no statistically significant difference was found in the availability of agreements and data-sharing practices across the 3 networks.

Data-sharing behaviors are influenced by various major factors, as indicated by the logistic regression study. One important effect was gender: women were less likely than men to share their datasets (OR 0.38, *P*=.015). The author believes that women may be encountering previous challenges in data sharing that could discourage them. This was also observed in another study where there was a significant interaction between gender and age, whereby there was a gender difference in confidence in privacy protection [44]. A professional group was also significant, with data managers and information and communication technology (ICT) exchanging datasets less frequently (OR 0.12, *P*=.012). Compared with other jobs, the researcher’s role was associated with a substantial decrease in student dataset sharing (OR 0.16, *P*=.034). Interestingly, years of research expertise had no significant impact on data-sharing behaviors. The study conducted by Piwowar [45] also indicates that few researchers are still sharing their research datasets. They only share their research datasets if they have experience in doing so. Also, the study shows that researchers in health subjects were least likely to share their datasets.

### Associated Factors Influencing Data Sharing

Data sharing has become an imperative in every sector. However, most organizations still maintain data silos, exercising unnecessary restrictions on data access and discouraging data sharing [46]. In times of COVID-19, disease surveillance and timely sharing of large digital datasets have taken center stage. For instance, as data sharing was deemed crucial for tackling the pandemic, the WHO and its partners established a global coalition on data sharing. The global coalition aimed to facilitate and promote effective, ethical, and equitable data sharing across geographies and disciplines for various purposes [47]. Furthermore, health research data sharing is being promoted to strengthen academic research, inform health policy and practice, and strengthen the integrity of the clinical trial system. The American politician and senior United States senator from Massachusetts, Elizabeth Warren, said

*When researchers have access to complete data, they can answer new questions, explore different lines of analysis, and more efficiently conduct large-scale analyses across trials*.

[Elizabeth Warren] [48]

The Senator also emphasized the need to publicly share data that produced null, inconclusive, or negative results. Data sharing has the potential to strengthen the practice of medical research.

The findings of this study contribute to the body of knowledge about health research data sharing in SSA. The results present experiences of health research data sharing from 15 African countries. It is interesting to note that slightly more than half of the respondents were using digital tools to collect research data from varied sources, including community and health facilities. Only about half (79/160, 49.4%) of the respondents indicated that they had digital research data management platforms, which means they lack essential foundations for enabling proper research data management and sharing.

### Access to Digital Research Platforms

Limited access to digital platforms for sharing research datasets was reported. The most common sharing was among colleagues within the same research institutions (53/169, 31.4%), while access to wider audiences, including policy makers at national and international levels, was significantly lower (19/169, 17.2% and 5/169, 3%, respectively). These results underscore the need for improved digital infrastructure and policies to enhance data accessibility. Studies from other regions highlight similar barriers to the widespread use of digital platforms for data dissemination.

### Factors Influencing Electronic Data Sharing

Several factors were identified as influencing data-sharing practices. Researchers with less experience (1‐5 y) were less likely to share datasets than those with more experience. This aligns with previous findings that indicate early-career researchers may face more barriers in data sharing, such as a lack of resources or networks. In addition, universities showed a higher likelihood of using digital research data management platforms (41/79, 51.9%) compared with research institutions, highlighting the disparity in digital capacity between different types of institutions.

The need to promote digital platforms for simplified research data management and sharing is evident, as about one-third of the respondents still use manual or paper-based tools. This may be due to limited knowledge and skills in using digital platforms, as well as having no or unreliable internet connections for uploading data [10,26,38,49]. The fear of data loss on digital platforms could also account for this. Furthermore, only about two-thirds were sharing research datasets. These findings correspond with the global trends in research data sharing, indicating that only 60% (n=2760) of more than 4600 Wiley authors from 112 countries participated in a survey conducted in 2016 and shared their research data and outputs [37].

Furthermore, in our study, some researchers did not share health research datasets (disaggregated anonymized or pseudonymized or analyzed data). Similarly, findings indicated that only 4% (n=119) of 2972 of the Directory of Open Access Repositories contain research datasets, while the rest host publications and related materials [50]. This could be due to researchers’ reluctance to share their research data with the public.

A study in Jordan reported that slightly more than half of the researchers (93/175, 53.1%) were unwilling to share their data with the public [51]. Another study reported that 80% (407/507) of researchers were willing to share their research data [52], while in another study, 54% (133/250) of the respondents used research repositories for data sharing [53]. In this study, most respondents indicated that they shared their research outputs through journal articles and scientific conferences, which may not be easily accessible to targeted users (eg, health practitioners and policy makers) for early translation to inform practice and policy. Interestingly, about one-third share their outputs through feedback meetings with the stakeholders.

Studies report diverse research data sharing experiences in LMICs [54]. Numerous challenges have been pointed out to hinder effective and efficient research data sharing and reuse in all economies [37,54,55]. Some of the challenges reported included limited privacy issues or protection, lack of regulations, heterogeneity and fragmentation of data sources, data storage limitations, incompatible data formats, political concerns, insufficient data sharing skills, and costs associated with data preparation and sharing [37,51,54].

Several factors influence stakeholders’ practices in sharing research data with different beneficiaries. These factors include the availability of robust digital platforms for data management and sharing, digital skills for data deposition and adaptation, incentives and motivations, organization support for data sharing, and legal and policy frameworks supporting data sharing. Furthermore, the willingness of researchers to contribute to ensuring the timely sharing of their research data [34,36,37].

There are, however, some challenges or barriers to the early sharing of research data or outputs for translation of research outputs. For example, reluctance to publish or share data was reported despite efforts for open data or open access, for example, journals emphasize data availability requirements when submitting their articles. Studies also reported challenges affecting the sharing of research data, including ethics and confidentiality concerns, fear of data misuse by people who had no or little contributions, lack of incentives and motivations, limited knowledge and skills for data management, and sharing through different platforms as well as limited financial support to data management and sharing. Furthermore, there are no clear guidelines and plans on health research data management, sharing, publications, authorships, and peer review of research datasets [44,53,54,56].

The findings reveal the need to develop research data governance skills, including acquiring suitable digital platforms for research data management and sharing, harmonizing data sharing requirements, and selecting and using repositories or platforms for data sharing. It is also imperative for research projects to develop data management plans that are in line with relevant national data governance or data protection and sharing legal frameworks, including data access and sharing agreements. Capacity building in health research data governance is important among researchers, research administrators, and members of ethics review committees. The capacity building mentioned here relates to training in data governance, covering aspects of data stewardship, protection, and sharing, as well as awareness of and compliance with existing data protection and privacy laws, including cross-border data sharing, as well as institutional policies and guidelines for data management and sharing [22,26].

### Maximizing the Benefits of and Minimizing the Risks of Sharing Health Research Data

Understanding the risks hindering health data sharing or access, Tiffin and colleagues [57] developed a digital health data governance framework in LMICs. The framework addresses 4 key domains to maximize the benefits of using health data while minimizing potential risks: ethical oversight, informed consent processes, data protection through data access controls, sustainability of fair data use, and application of relevant legislation [57].

Different studies have explored the benefits of sharing research data. For instance, data sharing allows or supports the following: replication and validation of research findings and reuse of the research data to answer new research questions, thus, cost savings [53]. Previous studies explored the attitudes and experiences of researchers on open access in LMICs, whereby there were variations in awareness and access to institutional repositories [52,58]. Furthermore, there are also inequalities between data-sharing practices among researchers in low-resource and high-resource settings [37,54].

Our study revealed positive attitudes toward openly sharing research data, including datasets. Similarly, this study reported practices in research data sharing but focused on health research data management and sharing health research datasets and publications through digital platforms. Another study focused on institutional repositories and indicated that they are mainly used to deposit and share research publications such as journal articles, reports, and dissertations [58].

### Limitations and Strengths

The findings of this study are self-reported; hence, they are likely to be overestimated for respondents who want to exaggerate their practices, thus expecting to be praised, or may underestimate their capacity, thus expecting extra support. We used web-based questionnaires to gather information from the respondents. This could have limited the opportunity for respondents to clarify any unclear questions or terms. For instance, there could be confusion between sharing research outputs such as disaggregated anonymized or pseudonymized datasets, analyzed data outputs, and other publications such as journal articles, theses, or reports.

The questionnaire pretesting stage ensured the survey questions were clear and understandable to the respondents. However, the possibility of misunderstandings was not completely eliminated. The web-based administration of questionnaires enables access to large and different populations and is an inexpensive, quick, and convenient approach to collecting data [40-42]. However, this approach has the disadvantage of having relatively lower response rates than face-to-face administration [40]. Nevertheless, in this study, the response rate was higher (75.9%) than the mean response rate (40.5%) for web-based data collection approaches reviewed by Blumenberg and Barros in 2018 [40].

We furthermore limited our exploratory study to quantitative responses on the use of digital platforms for sharing research data and the experience and practices of sharing the data. However, further insights are needed into what kinds of platforms are required, what it takes to share research data, and the facilitators and barriers to using appropriate digital research tools, ideally with a mixed method approach integrating quantitative and qualitative research data.

The study is confined to assessing the existence of research data sharing agreements and the influences on data sharing practices. It did not explore the contents or types of agreements or how they ought to be structured. Future studies should concentrate on the contents and types of agreements, particularly regarding the transfer and sharing of digital data.

However, this study has documented the extent of the use of digital platforms for research data management and sharing, and practices of sharing research datasets and publications in the health sector across SSA. The experiences gathered in this study may contribute to facilitating the adoption of sustainable and scalable digital platforms that have great potential to connect researchers, policy, and decision makers, and other relevant stakeholders to enable access to research outputs for facilitating the translation of research into policy and practice in SSA. Furthermore, the platforms should conform to users’ experiences and requirements and comply with national and subnational authorities’ project and research reporting requirements.

### Conclusion and Recommendations

The study presents an increased understanding of the existence and usage of digital research data platforms and stakeholders’ characteristics in the context of research data management and sharing. The study contributes further knowledge to using digital platforms to facilitate the early translation of research data and outputs to inform practice and policy, and reuse for research in the special context of SSA.

Most respondents used electronic data collection tools for research. Nearly half indicated they have a digital research data management platform and shared their research datasets and findings with the research community through various channels. Furthermore, many respondents shared their datasets and outputs via institutional repositories, scientific conferences, and journal articles. The study identified factors such as sex, professional category (health professionals, ICT specialists, and data managers), and role (researcher or student) influencing health research data sharing.

The results showed that an individual’s role had the strongest association with dataset sharing, followed by research experience, sex, and profession. In addition, logistic regression revealed that females were less likely to share their research datasets than males. Data managers and ICT professionals shared datasets less frequently within their professional group, while the researcher’s role was statistically significant in promoting data sharing.

Researchers and data management teams should be equipped with proper research data management platforms or data repositories to facilitate the sharing of research outputs. A research data governance and protection framework should be established and enforced to create an enabling environment for centralized research data repositories, curation, sharing, and reuse.

## Supplementary material

10.2196/69411 Multimedia Appendix 1 Questionnaire.

## Abbreviations

BMBF: Federal Ministry of Education and Research
cOR: crude odds ratio
FAIR: findable, accessible, interoperable, and reusable
ICT: information and communication technology
LMICs: low- and middle-income countries
NIMR: National Institute for Medical Research
OR: odds ratio
SSA: sub-Saharan Africa
WHO: World Health Organization
