# Supplementary material for: Experiences of Health Research Data Sharing Among Researchers in Sub-Saharan Africa: Cross-Sectional Study
Source: JMIR Form Res. 2025 Oct 23;9:e69411. doi: 10.2196/69411 (PMC12548969; doi:10.2196/69411)
Supplement: Multimedia Appendix 1 [file formative-v9-e69411-s001.docx]

**Appendix I: Questionnaire**

**Online Survey for assessing practices of and needs for digital research data sharing within five health innovation networks of sub-Saharan Africa**

**Section 1: Basic information and participant’s profile**

1. Name of the research network: *(select one answer only)*

o ANDEMIA o CEBHA+

o CYSTINET-Africa

o TAKeOFF

o TB Sequel

o Other, specify …………….

1. Your current country of residence: *(select one answer only)*

o Burkina Faso

o Cameroon

o DR Congo

o Ethiopia

o Germany

o Ghana

o Ivory Coast

o Malawi

o Mozambique

o Rwanda

o South Africa

o Tanzania

o The Gambia

o Uganda

o Zambia

o Other, specify …………….

1. Age in years ……………….
2. Sex: *(select one answer only)*

o Female

o Male

o Prefer not to mention

1. Highest level of education: *(select one answer only)*

o PhD

o Master

o Bachelor

o Diploma

o Certificate

o Other, specify ………………….

1. Your profession: *(select one answer only)*

o Computer Scientist

o Epidemiologist

o ICT Officer

o Laboratory Scientist

o Medical Doctor

o Nursing Officer

o Research Administrator

o Research Scientist

o Veterinary Doctor

o Other, specify ………………….

1. Your experience in research-related projects (in years) ………………
2. Your role in the research network: *(select one answer only)*

o Researcher

o Policy maker

o Health Practitioner

o Student supervisor

o Postdoctoral Student

o PhD Student

o Master Student

o Program/Project Manager

o Data Manager

o Lab Technologist

o ICT Officer

o Other, specify ………………….

**Section 2: Existing data/information systems and data sharing practices within the research project(s) that you are currently involved with in the context of your research network e.g. ANDEMIA**

**Sub-section 2.1: Overall research data management**

1. Please indicate which kind of diseases/themes your research studies are based on: ***(Tick all that apply)***

o Infectious diseases (IDs)

o Neglected tropical diseases (NTDs)

o Non-communicable diseases (NCDs)

o Environmental related diseases

o One Health (OH)

o Other, specify ………….

1. Please indicate which domain you are collecting research data on: ***(Tick all that apply)***

o Human health

o Animal health

o Environmental health

o Other, specify ……………….

1. Where do you collect your research data from? ***(Tick all that apply)***

o Human subjects/participants in communities (community-based study)

o Human subjects/participants in health facilities (hospital-based study)

o Records from health facilities including laboratories

o Animals (domestic/wild) in communities

o Animals (domestic/wild) in veterinary health facilities including laboratories

o Records from veterinary health facilities including records from veterinary research/wet laboratories

o Environmental samples from communities

o Environmental samples from health facilities

o Records from environmental health services

o Records from village offices (e.g. socio-demographic data of the population)

o Existing research/survey databases e.g. demographic health surveys

o Other, specify ………………

1. Which kind of research data do you collect? ***(Tick all that apply)***

o Human clinic-based data (e.g. medical history, physical examination)

o Human investigation-based data (e.g. lung function, x-ray, CT scan)

o Human laboratory-based data (e.g. blood, sputum, skin snip)

o Animal clinic-based data (e.g. physical examination)

o Animal investigation-based data (e.g. x-ray)

o Animal pathology-based data (e.g. carcass dissection)

o Epidemiological data (e.g. surveillance)

o Environmental data (e.g., climate-related data)

o Environmental laboratory-based data (e.g. vector or soil analysis)

o Secondary data from e.g. survey data bases

o Other, specify …………….

1. How do you collect your primary or secondary research data? ***(Tick all that apply)***

o Using paper-based forms

o Using electronic data collection tools (offline or online)

o Using both electronic data collection tools and paper-based forms

o Automatic data transfer from laboratory equipment into an electronic database

o Automatic transfer of data from radiological equipment e.g. digital x-rays, into an electronic database

o Extracting secondary data from online databases e.g. DHIS2

o Other, specify ………………….

**Sub-section 2.2: Using research data management platforms**

1. Which statistical software do you use for analysis/computations? ***(Tick all that apply)***

o SPSS

o SAS

o Stata

o Microsoft Excel

o EpiInfo

o R

o Python

o MAXQDA

o ATLAS.ti

o NVivo

o Other, specify …………

o I do not know

1. Do you have and use a digital platform/system(s) for research data management e.g. REDCap, OpenClinica, DSpace, DHIS2, OpenMRS? (If your answer is No or I do not know to Qn. 15, skip to Qn. 22)

o Yes, please indicate name:……………….

o No o I do not know

1. If your answer to Qn. 15 is Yes, please indicate how you rate your level of skills in using the digital research data platform:

o Very low

o Low

o Average

o High

o Very high

1. If your answer to Qn. 15 is Yes, please indicate which kind of research data you collect in your digital research data platform; (This is the same as Qn. 12, but this time referring to the digital research data platform): ***(Tick all that apply)***

o Human clinic-based data (e.g. medical history, physical examination)

o Human investigation-based data (e.g. lung function, x-ray, CT scan)

o Human laboratory-based data (e.g. blood, sputum, skin snip)

o Animal clinic-based data (e.g. physical examination)

o Animal investigation-based data (e.g. x-ray)

o Animal pathology-based data (e.g. carcass dissection)

o Epidemiological data (e.g. surveillance)

o Environmental data (e.g. climate related data)

o Environmental laboratory-based data (e.g. vector or soil analysis)

o Secondary data from e.g. survey data bases

o Other, specify …………….

1. If your answer to Qn. 15 is Yes, please indicate whether you created a data management plan for the digital research data platform:

o Yes

o No

1. If your answer to Qn. 15 is Yes, please indicate which formats of research data are collected in your digital research data platform: ***(Tick all that apply)***

o Text

o Numeric data

o Audios

o Images

o Videos

o Gene sequences (Genomic data)

o Geospatial data

o I do not know

o Other, specify …………….

1. If your answer to Qn. 15 is Yes, please indicate which features or functionalities are available in your digital research data platform: ***(Tick all that apply)***

o Data collection

o Data storage

o Data validation

o Data analyses

o Data visualization

o Data sharing

o Data transfer

o I do not know

o Other, specify …………….

1. If your answer to Qn. 15 is Yes, please indicate who has got access to your digital research data platform: ***(Tick all that apply)***

o Other researchers within your research institution e.g. NIMR

o Other researchers within your research network e.g. ANDEMIA

o Other researchers within the other four health innovation research networks

o Other researchers outside your own institution and the health innovation research networks

o Health professionals or practitioners in general

o Policy makers at subnational level

o Policy makers at national level

o Policy makers at international level

o Your research funder

o Other, specify ………….

**Sub-section 2.3: Dissemination of research data**

1. Would you agree to share your anonymised raw research datasets with any of the following before publication? Note: data sharing outside your own network should be covered by contracts (data sharing/transfer agreement). ***(Tick all that apply)***

o Other researchers within your research institution e.g. NIMR

o Other researchers within your research network e.g. ANDEMIA

o Other researchers in the other four health innovation research networks

o Other researchers outside your own institution and the health innovation research networks

o Health professionals or practitioners in general

o Policy makers at sub-national level

o Policy makers at national level

o Policy makers at international level

o Funding agencies

o The public sector

o The private sector

o I do not agree to share

o Other, specify ………….

1. Would you agree to share your research outputs (analysed / aggregated data) with any of the following before publication? ***(Tick all that apply)***

o Other researchers within your research institution e.g. NIMR

o Other researchers within your research network e.g. ANDEMIA

o Other researchers within the other four health innovation research networks

o Other researchers outside your own institution and the health innovation research networks

o Health professionals or practitioners in general

o Policy makers at subnational level

o Policy makers at national level

o Policy makers at international level

o Funding agencies

o The public sector

o The private sector

o I do not agree to share

o Other, specify ………….

1. Do you already share your research data via any of the below channels with a more public audience? ***(Tick all that apply)***

o Institutional data repository

o Funder data repository

o Discipline-specific data repository

o Journal data repository

o As supplementary material in a journal

o External repository or general-purpose data repository e.g. Figshare, Zenodo,

o Cloud file sharing e.g. Dropbox, Google Drive, Blog/website

o Other, specify …………….

o I do not share research data beyond my fellow researchers

1. Does your research project(s) have a data transfer/sharing agreement/policy with other partners? ***(Tick all that apply)***

o Partners of your own research network e.g. ANDEMIA

o Partners of any of the other four health innovation research networks

o Third party

o None

o I don’t know

o If you have ticked “third party” above, please specify ………….

1. Who are potential beneficiaries of your research findings with a focus on national and sub-national levels? ***(Tick all that apply)***

o Officials at the Ministry responsible for human health

o Officials at the Ministry responsible for animal health / livestock

o Officials at the Ministry responsible for agriculture

o Officials at the Ministry responsible for regional administration and local government

o Officials at other Ministries; please specify……….

o Zonal or Regional Health Offices o District or Council Health Offices

o Policy makers at international level

o Researchers in the other four health innovation research networks

o Researchers outside the other four health innovation research networks

o Health workers in the study settings

o Health workers in routine clinical care

o Community leaders in the study settings

o Community members in the study settings

o Clients/patients

o Funding agencies

o Regulatory bodies

o The public sector

o The private sector

o None of the above

o Other, specify ……………

1. How frequently do you share or disseminate your research findings for early translation of research findings?

o Never

o When opportunities arise

o Regularly as per project dissemination plan

o After completion of research study only

o Other, specify …………….

1. How do you share your research outputs for early translation of research findings? ***(Tick all that apply)***

o Project website

o Social media channels

o Newsletters

o Scientific conferences

o Journal articles and other publications

o Policy briefs

o Feedback meetings to policy makers

o Feedback meetings to health professionals

o Feedback meetings to community members

o Reports submitted to the ministry, region and/or district officials

o Other, specify …………….

**Section 3: Needs and requirements for data sharing platforms in general**

1. Do you think there is a need for a shared digital research data platform to facilitate early translation of research findings for policy and practice?

o Yes

o No

o Not sure

1. If your answer to Qn. 29 is No, what would be the possible reason(s) that would hinder your choice for the need for a shared digital research data platform? ***(Tick all that apply)***

o Confidentiality or data protection rights

o Agreements prohibiting data sharing

o Limitations in data security

o Lack of a shared data platform

o Lack of data protection policy and guidelines

o Lack of technological knowledge

o Lack of technological support

o Lack of collaboration between researchers and policy makers

o Potential violation of intellectual property rights

o Loss of research data ownership for scientific publications

o Costs of implementing a digital research data platform

o Lack of time to deposit data

o Other, specify …………

1. If your answer to Qn. 29 is Yes, who do you think should have access to the shared digital research data platform? ***(Tick all that apply)***

o Other researchers within your research institution e.g. NIMR

o Other researchers within your research network e.g. ANDEMIA

o Other researchers in the other four health innovation research networks

o Other researchers outside your own institution and the health innovation research networks

o Policy makers

o Health professionals or practitioners in general

o Other, specify………….

1. If your answer to Qn. 29 is Yes, what are your preferred data formats for sharing datasets in a digital research data platform? ***(Tick all that apply)***

o zip

o csv

o pdf

o xml

o html

o json

o geojson

o excel format: xls, xlsx

o rss feed

o other, specify ………

o I don’t know

1. If your answer to Qn. 29 is Yes, what is your preferred data category for datasets to be available in a shared digital research data platform?

o Anonymized individual/client-level data

o Aggregated data

o Both (individual level data and aggregated data)

1. If your answer to Qn. 29 is Yes, what features would you like to be included in a shared research data platform? ***(Tick all that apply)***

o Collection of data electronically, both in online and offline modes

o Capturing multimedia data formats

o Data cleaning, including validation

o Ensuring data integrity

o Management of metadata (data descriptions)

o Data analysis and visualisation

o Archiving of data

o Sharing of data o Strong security and authentication mechanisms

o Supporting unlimited file types

o Free and open-source platform

o Integration and interoperability (data exchange with other systems)

o Supporting multiple languages

o Supporting the FAIR (Findable, Accessible, Interoperable, and Reusable) Data Principles

o Other, specify …………..

1. If your answer to Qn. 29 is Yes and if you are working with infectious diseases, what additional features would you like to be included in a shared digital research data platform?

…………………………………………………………….…………………………………………………………….

1. If your answer to Qn. 29 is Yes, and if you are working with neglected tropical diseases, what features would you like to be included in a shared digital research data platform?

…………………………………………………………….…………………………………………………………….

1. If your answer to Qn. 29 is Yes and if you are working with non-communicable diseases, what features would you like to be included in a shared digital research data platform?

…………………………………………………………….…………………………………………………………….

1. If your answer to Qn. 29 is Yes, and if you are working with One Health, what features would you like to be included in a shared digital research data platform?

…………………………………………………………….…………………………………………………………….
